# Malignant Melanoma as a Rare Cause of Penile Cancer: A Case Report

**DOI:** 10.7759/cureus.104081

**Published:** 2026-02-22

**Authors:** Diego Guarda, Gerardo Bascuñán, Priscilla Márquez, Diego I Mendez-Villanueva

**Affiliations:** 1 General Medicine, Clínica Andes Salud, Puerto Montt, CHL; 2 General Medicine, Hospital Familiar y Comunitario de Carahue, Carahue, CHL; 3 General Medicine, Universidad Andres Bello, Santiago, CHL; 4 Dermatology, Universidad de Santiago de Chile, Santiago, CHL

**Keywords:** malignant melanoma, mucosal melanoma, penile cancer, penile melanoma, rare genitourinary tumors

## Abstract

Malignant melanoma is a neoplasm originating from melanocytes and may arise at cutaneous or extracutaneous sites. Penile involvement is uncommon and may present with variable clinical manifestations. We describe a case of a 47-year-old male who presented with a progressively enlarging lesion involving the glans penis, initially associated with pruritus and later with severe local pain and bilateral inguinal lymphadenopathy. Histopathological examination of an incisional biopsy confirmed malignant melanoma of the penis. Imaging studies performed for staging demonstrated regional lymph node involvement and disseminated visceral disease. The patient underwent surgical management followed by systemic treatment after multidisciplinary evaluation. This case illustrates the clinical presentation, diagnostic approach, and management of penile malignant melanoma.

## Introduction

Melanoma is a malignant neoplasm arising from melanocytes, accounting for approximately 1-3% of all malignancies worldwide, yet responsible for the majority of skin cancer-related deaths due to its aggressive biological behavior and high metastatic potential [1]. Although melanoma most commonly arises in the skin, it can also originate from melanocytes in mucosal surfaces, the uveal tract, and other extracutaneous sites [2].

The global incidence of melanoma has steadily increased over recent decades, particularly among fair-skinned populations, with ultraviolet radiation exposure being the most significant environmental risk factor for cutaneous forms [3]. Other recognized risk factors include genetic susceptibility, family history, the presence of dysplastic nevi, immunosuppression, and increasing age [4]. Despite advances in early detection and treatment, advanced-stage melanoma continues to carry a poor prognosis, especially when regional lymph nodes or distant organs are involved [5].

Mucosal melanomas represent a rare subset, accounting for approximately 1-2% of all melanoma cases, and are biologically distinct from cutaneous melanoma [6]. They are characterized by a more aggressive clinical course, later stage at diagnosis, and worse overall survival, largely due to delayed recognition and lack of early visible symptoms [7]. Common mucosal sites include the head and neck region, anorectal area, female genital tract, and, less frequently, the male genitourinary system [8].

Penile melanoma is an exceedingly rare malignancy, comprising less than 1% of all primary penile cancers and less than 0.2% of all melanomas in men [9]. It typically arises from melanocytes within the mucosa of the glans penis, foreskin, or urethral meatus [10]. Due to its rarity and non-specific initial presentation, penile melanoma is frequently diagnosed at advanced stages, contributing to its poor prognosis [11]. Clinically, it may present as a pigmented or non-pigmented lesion with asymmetry, irregular borders, ulceration, bleeding, or rapid growth; symptoms such as pain, pruritus, or discharge may occur but are often absent early in the disease course [12]. Regional lymphatic spread, particularly to the inguinal lymph nodes, is common and represents a major prognostic factor [13].

There is no standardized treatment protocol for penile melanoma due to its low incidence; however, surgical resection remains the cornerstone of management, frequently combined with inguinal lymphadenectomy in cases of nodal involvement [14]. Recent advances in immunotherapy, particularly immune checkpoint inhibitors, have improved outcomes in advanced melanoma and are increasingly incorporated into the management of mucosal and genital melanomas [15]. Given its aggressive behavior and diagnostic challenges, increased clinical awareness and early biopsy of suspicious penile lesions are essential to improve survival and reduce disease-related morbidity [16].

This case report highlighted the rapid clinical progression, early metastatic dissemination, and diagnostic challenges of penile malignant melanoma in a relatively young patient, emphasizing the importance of early recognition and multidisciplinary management.

## Case presentation

A 47-year-old male presented to a primary care clinic with a four-month history of a progressively enlarging lesion located at the base of the glans penis. The lesion was initially pruritic and progressively enlarged, eventually involving the entire surface of the glans penis. The patient also reported unquantified weight loss and progressive local pain.

**Figure 1:**
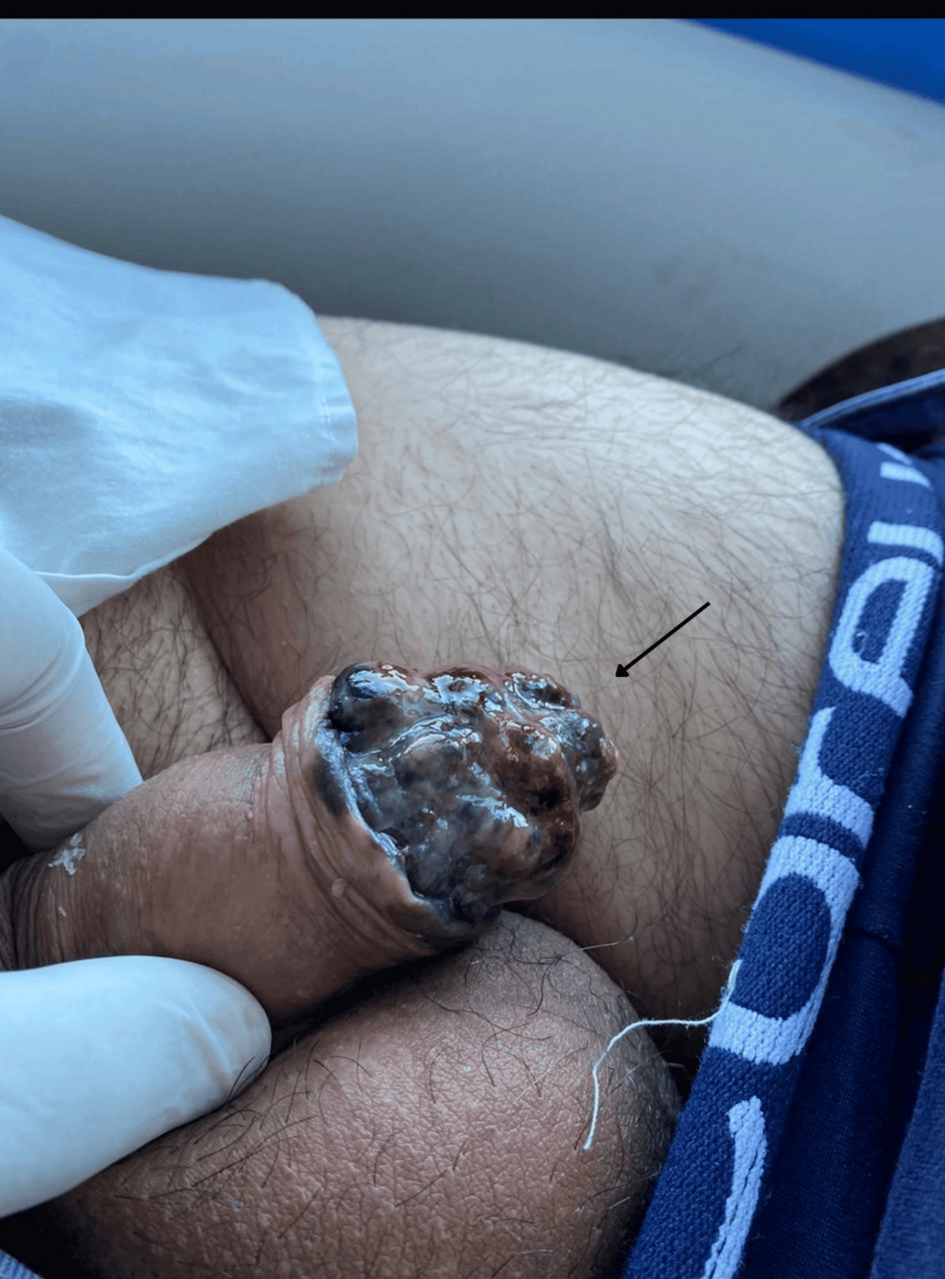
Advanced penile malignant melanoma involving the glans. Clinical photograph showing a large exophytic, ulcerated, nodular lesion involving the glans penis, with heterogeneous pigmentation, irregular borders, and areas of necrosis and hemorrhage. The lesion exhibits asymmetric growth and destructive local morphology, features consistent with advanced-stage penile malignant melanoma. The arrow highlights the primary malignant lesion.

Physical examination revealed a complex, raised, mammillated, indurated, and irregular lesion involving the glans penis, with areas of ulceration and purulent local secretion (Figure 1). No urethral discharge was observed. The patient reported severe pain at rest, exacerbated by palpation. Bilateral indurated inguinal lymphadenopathy was noted. Dermoscopy was not performed during the initial evaluation, as the patient was assessed in a primary care setting without access to dermoscopic equipment. Initial differential diagnoses included benign pigmented lesions, penile squamous cell carcinoma, and infectious or inflammatory conditions.

An initial biopsy of the balanopreputial sulcus demonstrated an ulcerated malignant melanoma of the superficial spreading type, in a vertical growth phase, with invasion into the lamina propria. Histopathological analysis revealed a Breslow thickness of 2.5 mm, a mitotic rate of 6 mitoses/mm^2^, positive lymphovascular invasion, scarce tumor-infiltrating lymphocytes, and positive lateral surgical margins. The lesion was staged as pT3b according to the American Joint Committee on Cancer (AJCC) eighth edition.

**Figure 2:**
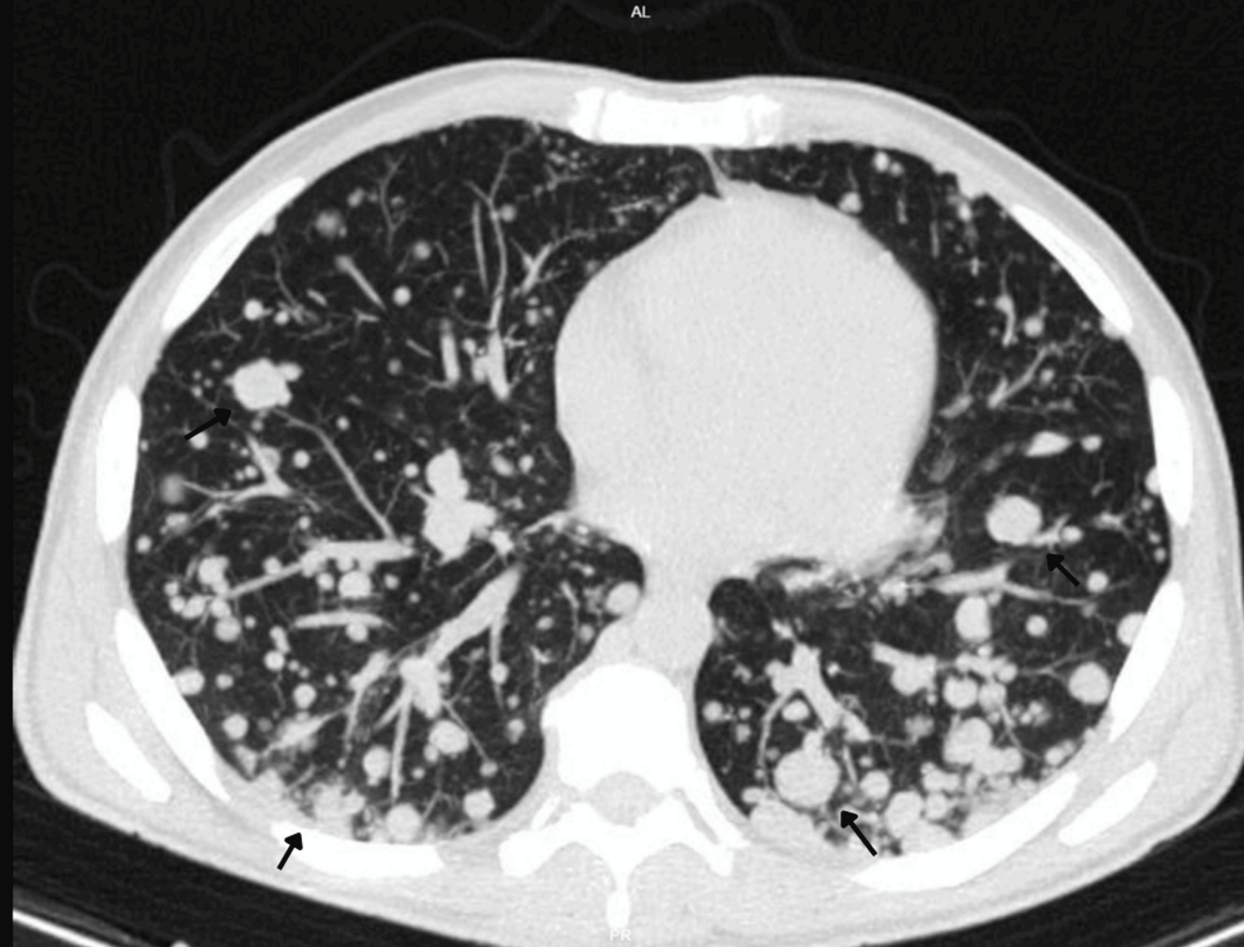
Contrast-enhanced CT of the chest, axial plane. Axial CT image demonstrates numerous bilateral pulmonary nodules of variable size, diffusely distributed throughout both lung fields, several of which show cavitation, consistent with widespread pulmonary metastatic disease. Arrows highlight representative pulmonary metastatic nodules. CT: computed tomography

**Figure 3:**
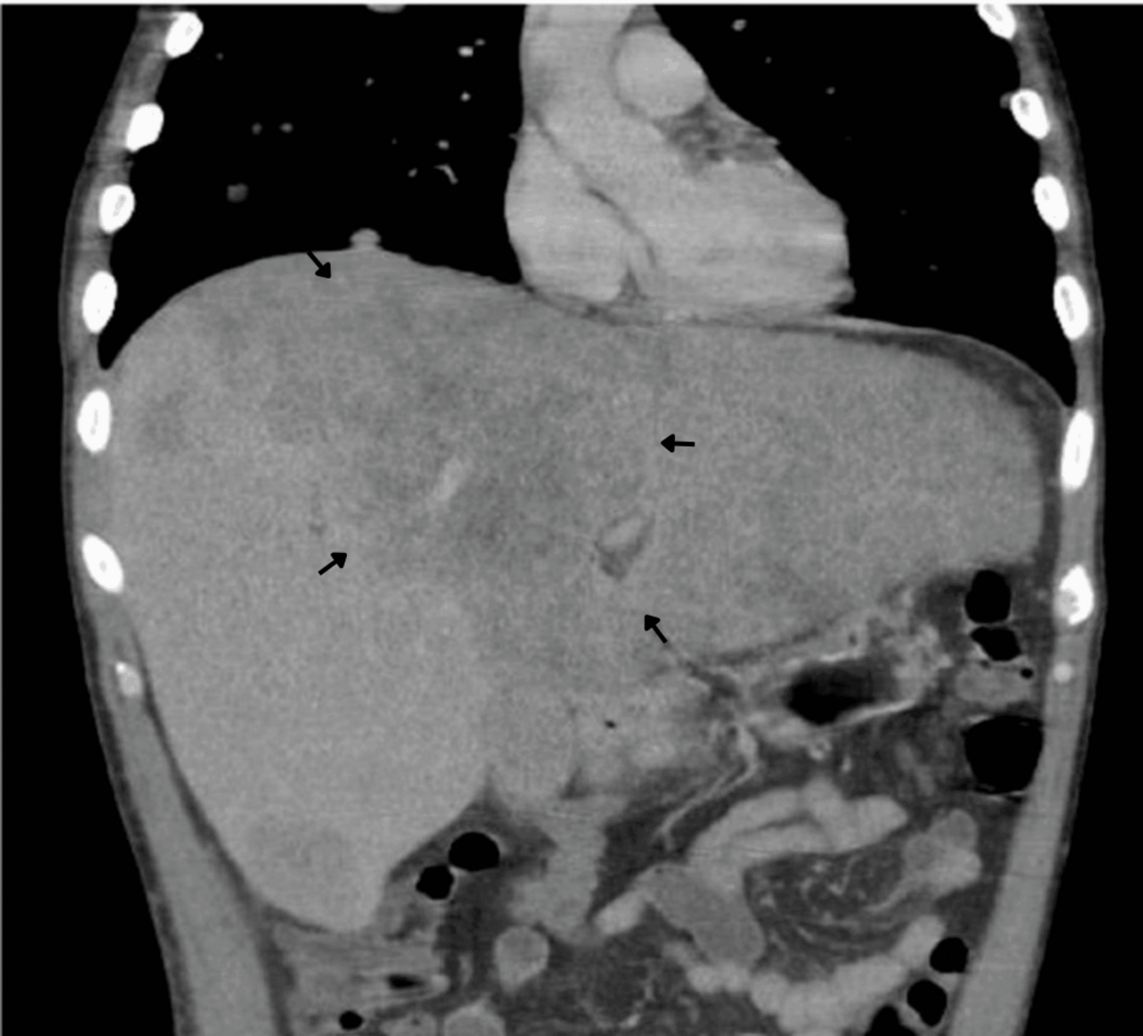
Contrast-enhanced CT of the abdomen, coronal plane. Coronal CT image reveals hepatomegaly with diffuse infiltrative involvement of both hepatic lobes. A large coalescent mass replaces most of the left hepatic lobe, findings consistent with extensive hepatic metastases. Arrows delineate the coalescent metastatic hepatic mass. CT: computed tomography

**Figure 4:**
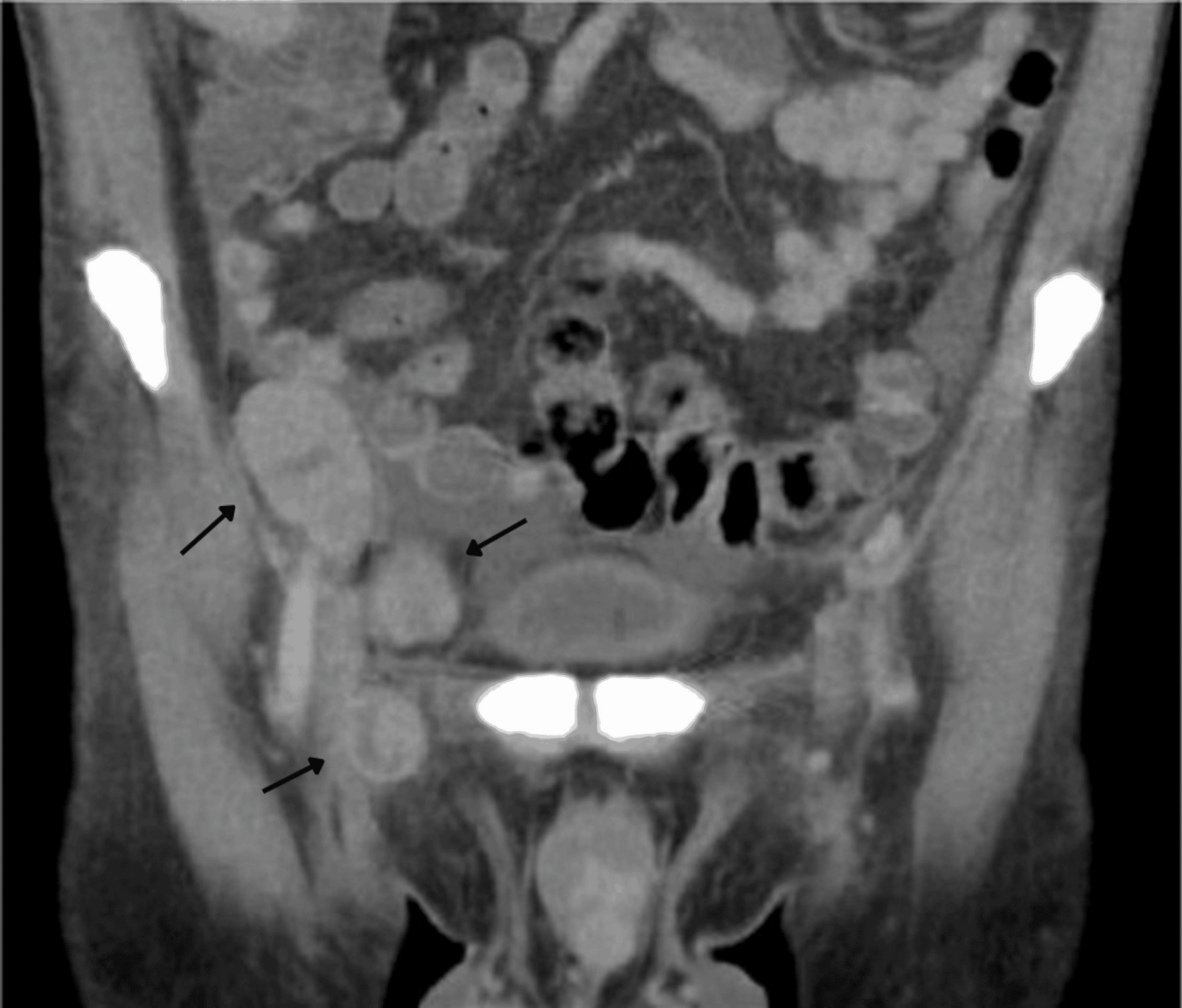
Contrast-enhanced CT of the pelvis, coronal plane. Coronal CT image demonstrates multiple enlarged bilateral inguinal and iliac lymph nodes with rounded morphology, consistent with metastatic lymphadenopathy in the context of advanced malignant melanoma. Arrows highlight metastatic lymphadenopathy. CT: computed tomography

**Figure 5:**
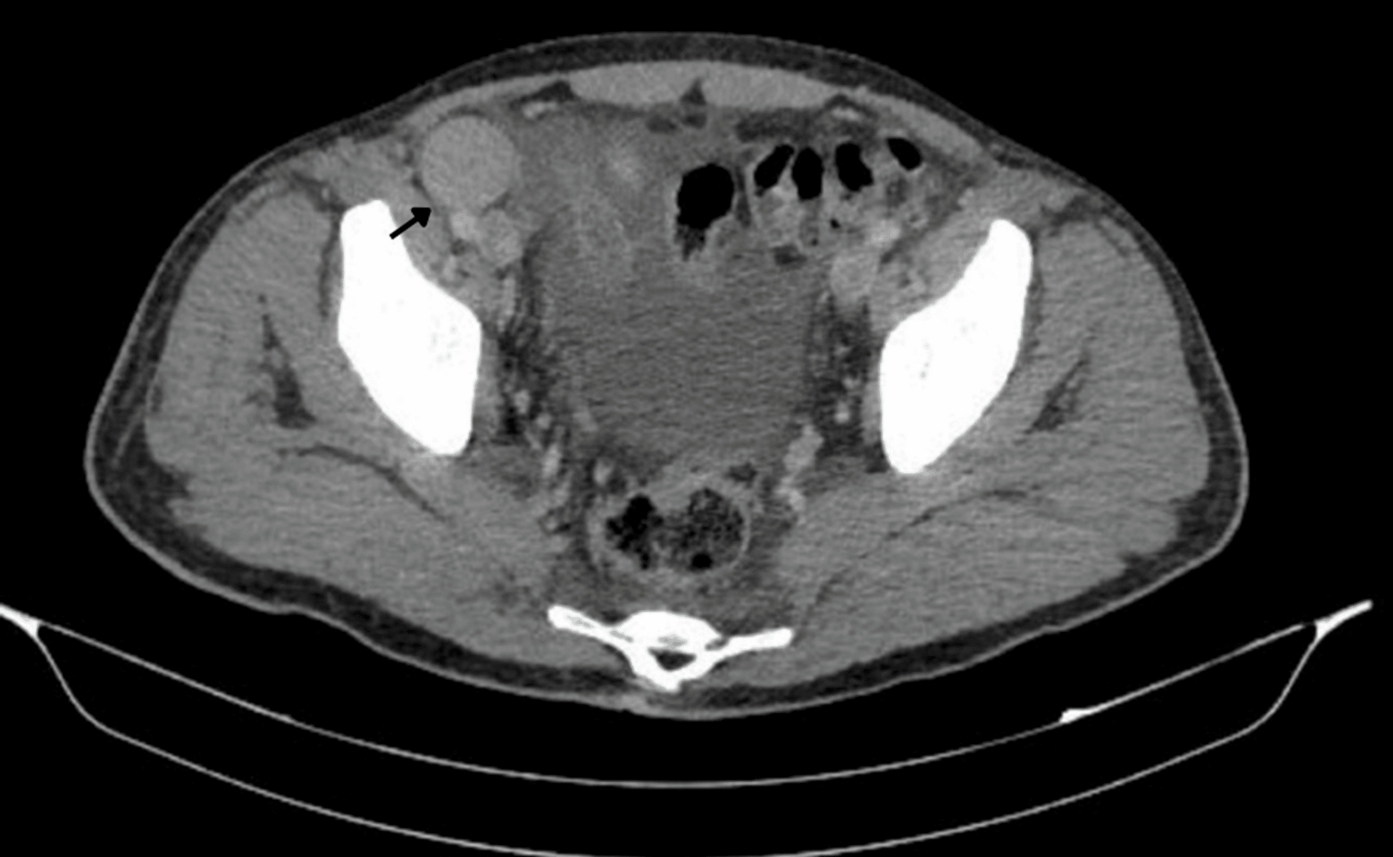
Contrast-enhanced CT of the pelvis, axial plane. Axial CT image of the pelvis demonstrating enlarged iliac and inguinal lymph nodes. The arrow indicates pathological lymphadenopathy consistent with metastatic involvement. CT: computed tomography

For staging and assessment of systemic disease, a contrast-enhanced computed tomography (CT) scan of the chest, abdomen, and pelvis was performed. Imaging demonstrated extensive metastatic disease, including multiple bilateral pulmonary nodules, several of them cavitated (Figures 2), mediastinal and hilar lymphadenopathy measuring up to 28 mm, diffuse hepatic infiltration with a large coalescent mass replacing the left hepatic lobe, measuring approximately 26×14 cm (Figure 3), as well as retroperitoneal, iliac, and bilateral inguinal lymphadenopathy (Figures 4, 5). Additional findings included splenomegaly and minimal ascites. Overall, these findings were consistent with widespread metastatic melanoma involving both nodal and visceral compartments.

Given the extent of local tumor involvement, the patient subsequently underwent total penectomy. Definitive histopathological examination of the surgical specimen revealed an ulcerated superficial spreading melanoma with vertical growth phase, infiltrating the corpus spongiosum, corpora cavernosa, and urethral meatus. The tumor demonstrated a maximum Breslow thickness of 12 mm, Clark Level V invasion, a high mitotic index (21 mitoses/mm^2^), lymphovascular and perineural invasion, and the presence of satellite metastases within the corpus cavernosum. Tumor-infiltrating lymphocytes were scarce. Surgical margins were free of invasive and in situ melanoma, with distances greater than 30 mm. The pathological stage was pT4b (AJCC eighth edition). The change from the initial pT3b staging to pT4b reflected deeper tumor invasion identified in the definitive surgical specimen, including involvement of adjacent anatomical structures not fully appreciated in the initial biopsy.

Molecular testing for BRAF mutation was attempted using the Idylla Platform (Mechelen, Belgium: Biocartis); however, the analysis was inconclusive due to insufficient DNA in one sample and the absence of BRAF. The inconclusive BRAF result limited the possibility of targeted therapy selection and supported the decision to pursue systemic immunotherapy.

Subsequently, the patient underwent bilateral inguinal lymphadenectomy. Histopathological evaluation revealed metastatic melanoma in two of 17 left inguinal lymph nodes and three of 18 right inguinal lymph nodes, with the largest metastatic deposit measuring over 19 mm and evidence of extranodal extension. These findings confirmed regional nodal metastatic disease. Key clinicopathological and staging findings are summarized in Table 1.

**Table 1:** Summary of key clinicopathological and staging findings. Summary of key clinical, histopathological, imaging, and staging findings throughout the patient’s disease course. The table integrates initial biopsy results, imaging-based staging, definitive surgical pathology, and lymph node involvement to illustrate the progression from localized penile malignant melanoma to advanced metastatic disease. AJCC: American Joint Committee on Cancer Idylla Platform (Mechelen, Belgium: Biocartis)

Domain	Findings
Patient and timeline	Forty-seven-year-old male; ~4-month progression of glans lesion; pruritus → progressive pain; unquantified weight loss
Primary lesion (clinical examination)	Raised, mammillated, indurated, irregular glans lesion with ulceration and purulent local secretion; severe pain; bilateral indurated inguinal lymphadenopathy
Initial biopsy (balanopreputial sulcus)	Ulcerated malignant melanoma, superficial spreading type; vertical growth phase; invasion into lamina propria; Breslow 2.5 mm; mitotic rate 6 mitoses/mm^2^; lymphovascular invasion positive; tumor-infiltrating lymphocytes scarce; lateral surgical margin positive; pT3b (AJCC eighth edition)
Staging CT (chest/abdomen/pelvis, contrast-enhanced)	Multiple bilateral pulmonary nodules (several cavitated); mediastinal/hilar lymphadenopathy up to 28 mm; diffuse hepatic infiltration with large coalescent mass replacing left hepatic lobe (~26×14 cm); retroperitoneal, iliac, and bilateral inguinal lymphadenopathy; splenomegaly; minimal ascites
Penectomy specimen (definitive pathology)	Ulcerated superficial spreading melanoma; vertical growth phase; infiltration of corpus spongiosum, corpora cavernosa, and urethral meatus; maximum Breslow 12 mm; Clark Level V; mitotic index 21 mitoses/mm^2^; lymphovascular and perineural invasion present; satellite metastases within corpus cavernosum; tumor-infiltrating lymphocytes scarce; margins free of invasive/in situ melanoma (>30 mm); pT4b (AJCC eighth edition)
Molecular testing	BRAF testing attempted (Idylla); inconclusive due to insufficient DNA in one sample and absence of BRAF
Inguinal lymphadenectomy	Metastatic melanoma in 2/17 left inguinal nodes and 3/18 right inguinal nodes; largest metastatic deposit >19 mm; extranodal extension present

The patient was referred for systemic oncologic management and initiated multidisciplinary follow-up. Despite treatment planning, the clinical course remained aggressive, and the patient died approximately one year after diagnosis due to disease progression.

## Discussion

Penile malignant melanoma represents a rare and aggressive subtype of mucosal melanoma, characterized by a high propensity for early regional and distant metastasis. In previously published series, most patients are diagnosed at advanced stages, frequently with regional lymph node involvement at presentation, which remains the most important prognostic determinant of survival [9,13,14].

The clinical presentation in our patient is consistent with prior reports describing rapidly progressive, ulcerated, and symptomatic lesions involving the glans penis [9,11,12]. Similar to other published series, delayed presentation and advanced local disease were evident, likely reflecting both the rarity of the condition and the tendency for patients to postpone medical evaluation of genital lesions [14]. In our case, the presence of early nodal involvement and rapid progression to widespread visceral metastases underscores the aggressive biological behavior described in mucosal melanomas compared to their cutaneous counterparts [6-8].

Histopathological features observed in this case, including deep invasion, high mitotic index, lymphovascular and perineural invasion, and satellite metastases, have all been associated with poor prognosis in penile melanoma and mucosal melanoma in general [5,7,14]. The progression from an initial pT3 lesion to a pT4 tumor with extensive metastatic spread further illustrates the rapid disease evolution reported in previous institutional experiences [9,14].

Surgical resection remains the cornerstone of management in penile melanoma, with partial or total penectomy recommended depending on tumor extent, and regional lymphadenectomy indicated in the presence of nodal disease [10,14]. In advanced-stage disease, systemic therapy plays a critical role. Recent evidence supports the use of immune checkpoint inhibitors, either as monotherapy or in combination, in patients with mucosal melanoma, offering improved disease control and survival compared to historical outcomes [15].

This case reinforces existing literature emphasizing that early recognition and prompt biopsy of suspicious penile lesions are essential to avoid advanced-stage presentation. Given the absence of standardized screening strategies and the rarity of the disease, heightened clinical awareness among primary care physicians, urologists, and dermatologists remains fundamental to improving outcomes in this uncommon but life-threatening malignancy.

## Conclusions

Penile malignant melanoma is a rare but highly aggressive malignancy, frequently associated with delayed diagnosis, early regional lymphatic spread, and poor prognosis. This case illustrates the rapid clinical progression and extensive metastatic potential of this tumor, emphasizing the need for heightened clinical awareness. Clinicians should maintain a high index of suspicion when evaluating rapidly evolving, pigmented, ulcerated, or symptomatic penile lesions, even in younger patients.

Early recognition, prompt histopathological confirmation, and accurate staging are essential to guide appropriate therapeutic decision-making. Management requires a multidisciplinary approach involving urology, oncology, pathology, and radiology, particularly in advanced cases. Increased awareness and early intervention remain key factors in improving outcomes for patients with this uncommon but life-threatening disease.
